# The Effect of Copper and Copper Oxide Nanoparticles on Rainbow Trout (*Oncorhynchus mykiss* W.) Spermatozoa Motility after Incubation with Contaminants

**DOI:** 10.3390/ijerph19148486

**Published:** 2022-07-12

**Authors:** Małgorzata Garncarek, Katarzyna Dziewulska, Monika Kowalska-Góralska

**Affiliations:** 1 Institute of Biology, Doctoral School, University of Szczecin, 70-383 Szczecin, Poland; malgorzata.garncarek@phd.usz.edu.pl; 2 Department of Hydrobiology, Institute of Biology, University of Szczecin, 71-412 Szczecin, Poland; 3 Department of Limnology and Fishery, Institute of Animal Breeding, Faculty of Biology and Animal Science, Wrocław University of Environmental and Life Sciences, 51-630 Wroclaw, Poland; monika.kowalska-goralska@upwr.edu.pl

**Keywords:** nano, copper, spermatozoa, CASA, fish, *Oncorhynchus mykiss*, rainbow trout

## Abstract

The study aimed to analyse the effect of copper nanoparticles of similar particle size of Cu and CuO and copper ions (CuSO_4_) on the motility parameters of rainbow trout spermatozoa after long-term exposure and compare its harmful effect. Nanoproducts of Cu and CuO (Cu NPs, CuO NPs) of primary particle size around 50 nm and ionic solution of CuSO_4_ were used for the study. Suspension of concentrations 0, 1, 5, 10, 25, and 50 mg Cu·L^−1^ of Cu NPs, CuO NPs, and CuSO_4_ was dissolved in an artificial seminal plasma. Milt was mixed with the prepared solution and stored in a fridge, at 6 °C, for 96 h. At the defined incubation time, spermatozoa were activated for movement, and six motility parameters were evaluated using an automated system (CASA). Increasing concentrations of Cu NPs, CuO NPs, and CuSO_4_ in an incubation medium in parallel decreased the percentage of motile sperm (MOT). The effect of Cu NPs and ionic copper on MOT was more deleterious than that of CuO NPs. Copper products slightly increased the velocity (VCL) compared to the control, particularly up to 24 h of storage. Linearity (LIN) was improved by three tested products, particularly CuO NPs. Generally, the motility duration was prolonged when the sperm was incubated with copper products compared to the control. Nanoproducts made from different compounds of the same elements of similar particle size have a different effect on cells. Cu NPs were more harmful than CuO NPs. The effect of Cu NPs was similar to an ionic form of CuSO_4_. When incubated, the copper nanoproducts and ionic form exert a slightly positive effect on spermatozoa velocity, linearity, and motility duration, particularly up to 24 h of storage.

## 1. Introduction

The presence of substances in water, which are not produced naturally by biologically active species is of great concern to the public. Many of these so-called xenobiotic chemicals have been found to be harmful, carcinogenic, or capable of accumulating in the environment and having toxic impacts on ecosystems. One of such chemicals includes nanoparticles. Previous studies indicate that the sensitivity of organisms to nanoparticle contamination varies [1,2,3]. Possible effects of nanoproducts on the organism are: adhesion to a cell’s surface, accumulation in tissues, transformation into an ionic form, causing dysfunction of metabolic pathways, disturbance of mitochondrial membrane potential, and oxidative stress [4,5,6]. In extreme cases, nanoparticles’ toxic effects may cause the death of organisms [7,8,9,10]. In animals with external fertilisation, nanoparticles not only have a direct effect on organisms but also on gametes discarded into the aquatic environment. The composition of the aquatic environment affects the mechanism triggering activation of gametes, movement characteristics of sperm, and ability to fertilise [11,12,13,14]. In this environment, the damaging effects of the nanoparticles on gametes may impair or prevent the reproduction of organisms.

Copper as one of the microelements in small doses is desirable in the environment for the proper functioning of organisms. However, many elements in excessive doses may have a harmful or even toxic effect [15]. Nanometals are less harmful than ionic metals [16,17,18,19,20]. Some studies have found the opposite effect [5,21]. The lethal dose of copper nanoproducts was determined for several model organisms [19,21,22,23]. The dose varies depending on the compound and species (Table 1).

Due to their antibacterial and antifungal effects [31,32], the number of products containing nanoproducts is constantly increasing [33]. Consequently, the amount of nanoparticles released into the environment represents an increasing risk to organisms [34,35,36,37,38]. Studies on the toxicity of nanoparticles to cells and organisms are necessary to know how to protect them. Establishing standards and environmental protection principles becomes an urgent task.

Few tests have been conducted on fish. The mortality of LC_50_ has been reported in the case of zebra fish (*Danio rerio*) of 1.5 mg·L^−1^ Cu NPs after 48 h of incubation [39] and rainbow trout (*Oncorhynchus mykiss*) after 10 days of exposure to low concentrations of Cu NPs—mortality of 19% in 100 µg L^−1^ of Cu NPs [38]. Fewer studies were conducted on the effect on the cells and their metabolism. Spermatozoa as a cell is a good model for studying the ecotoxic effects of nanoproducts and other chemicals on cells [40,41,42,43,44]. There are few studies on the effect of NPs on fish spermatozoa. Ozgur et al. [45,46,47,48] studied the effect of several nanoparticles including ZnO, TiO_2_, SiO_2_, and Fe_3_O_4_ NPs on carp and rainbow trout spermatozoa motility parameters after 1 or 3–4 h of incubation with nanoproducts. There are two methods of studying the effects of pollutants on sperm: storage of immotile spermatozoa with pollutants for defined time periods (long-term contact), or direct interaction by placing the nanoproduct into an activation media that triggers motility (short-lived contact during motility phase) [41,49,50,51,52,53,54]. In the first model, higher concentrations must be used to obtain a detrimental effect. There is little data on the effects of nanoproducts made of different compounds of one the same element on biological objects. The direct effect of two copper nanoproducts on spermatozoa motility was previously investigated by our group. A more harmful effect of ionic form than nanoparticles was obtained in the study while diverse effects of the nanoproducts were not clear [23]. The aim of this study was to use long-term storage to differentiate the effect of two copper nanoproducts, Cu NPs and CuO NPs, on rainbow trout spermatozoa motility parameters.

## 2. Materials and Methods

### 2.1. Milt Collection

In the middle of the reproduction season, during artificial spawning, sperm from ten farmed rainbow trout (*Oncorhynchus mykiss* W.) was stripped by employees of The Trout Breeding Center “Kuźniczka” in Wieleń (52°57′03,8232″ N 16°13′52,0320″ E) into plastic containers. Sperm was transported to the laboratory in plastic containers on ice (2–4 °C) for about 2 h. The samples of the best quality of milt (motility > 80%) were selected for experiments based on computer-assisted sperm analysis (CASA).

### 2.2. Chemicals and Storage Condition

The copper nanoproducts (NPs) of Cu NPs and CuO NPs of similar primary particle size of around 50 nm and ionic form CuSO_4_ were purchased from Sigma Aldrich. The basic suspension of compounds 1 g Cu·L^−1^ was prepared in artificial seminal plasma (ASP: 125 mM NaCl, 40 mM KCl, 1 mM CaCl_2_, 1 mM MgCl_2_, 50 mM Tris, pH 8.5, 363 mOsm·kg^−1^, composition by Morisawa and Morisawa [55] modified by Dziewulska) and sonicated in an ultrasonic bath for 30 min. Following stock suspension: 0, 1, 5, 10, 25, 50 mg Cu·L^−1^ of testing compounds were prepared by mixing in artificial seminal plasma. Four milt samples were mixed individually with artificial seminal plasma containing an adequate nanoproduct concentration in the ratio 1:10 and stored in thin layers (5 mm), at 6 °C, for 96 h. The samples were mixed every 12 h. At defined incubation times: 0, 2, 12, 24, 48, 72, and 96 h activation of spermatozoa motility was determined in CASA following the method used in Kowalska-Góralska et al. [23].

### 2.3. Computer-Assisted Sperm Analysis (CASA)

Spermatozoan motility parameters were determined using an automated system, the Sperm Class Analyzer (SCA) v. 4.0.0 manufactured by Microptic S.L., Barcelona, Spain. At 10 s after activation in distilled buffered water with 20 mM Tris, pH 9.0, at 8 °C, motility parameters were recorded following the method used in Kowalska-Góralska et al. [23]. The temperature of the activating solution was maintained at 8 °C using a cooling block (FINEPCR, Seoul, Korea), and the microscope table was equipped with a cooling device (Semic Bioelektronika, Kraków, Poland.) Milt suspension in ASP with copper products (1–2 μL) was added to 100 μL of activating solution, in a 1.5 mL in a polyethene Eppendorf tube and mixed. Six parameters characterising motility were chosen for analysis: 1. MOT—the percentage of motile spermatozoa (%) (where the criterion of motility was an average path velocity VAP > 20 µm·s^−1^), 2. VCL—curvilinear velocity (µm·s^−1^), 3. LIN—linearity (%), 4. ALH—amplitude of lateral head displacement (µm), 5. BCF –the track crossing frequencies, 6. MD—motility duration (s).

### 2.4. Statistical Analysis

Two-way repeated measure of ANOVA was used to test the effects of (1) Cu NPs, CuO NPs, and CuSO_4_ and (2) time of incubation upon measured motility parameters. NIR test was used for all subsequent posthoc comparisons. All statistical procedures were performed with Statistica 13.1 software, and the results were regarded as statistically significant at the level of 0.05.

## 3. Results

### 3.1. Percentage of Motile Spermatozoa (MOT)

**Figure 1:**
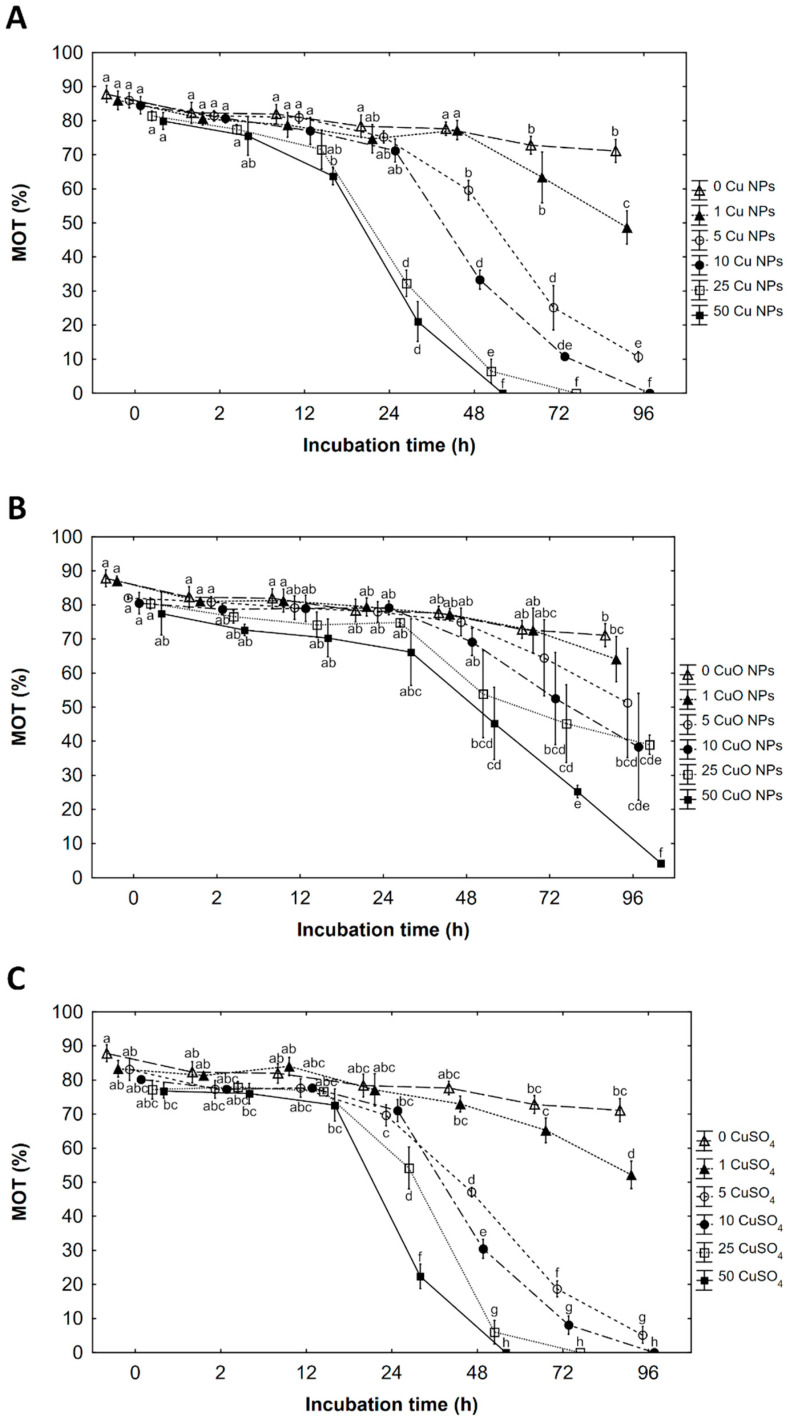
The effects on percentage of motile spermatozoa (MOT) of copper as (**A**) Cu nanoparticles (Cu NPs), (**B**) CuO nanoparticles (CuO NPs), and (**C**) ions of CuSO_4_ solution at concentration: 0, 1, 5, 10, 25 and 50 mg copper·L^−1^ after a defined time of incubation 0, 2, 12, 24, 72 and 96 hours (h). Sperm of rainbow trout (*Oncorhynchus mykiss* W.) was mixed with incubation solution in the of ratio 1:10. Values marked with the same letter are not significantly different from one another (*p* > 0.05). Two-way or one-way ANOVAs and NIR tests were used for the post-hoc comparison. Mean value ± SEM.

A highly significant interaction was found between Cu NPs, CuO NPs, and CuSO_4_ and incubation time for the MOT (Table 2). Tested factors reduced the percentage of motile sperm. In all of them, slight decrease in motility was noticed already at 0 time of incubation when spermatozoa motility was traced after temporary contact of gametes during preparing working solution then activated with water. In incubation with Cu NPs the earliest significant decrease in MOT was observed after 12 h of incubation at 50 mg Cu·L^−1^ as Cu NPs compared to the control, then constantly decreased across the storage. IC_50_ (immobilization) occurred in the concentration after 24 h of incubation. In 25 mg Cu·L^−1^ as Cu NPs, a decrease in MOT occurred after 24 h of incubation, while in 5 and 10 mg Cu·L^−1^ after 48 h. After 96 h of incubation, a significant decrease in MOT was observed at 1 mg Cu·L^−1^. Cessation of movement IC_100_ was detected in 48, 72, and 96 h of incubation in a solution of 50, 25, and 10 mg Cu·L^−1^ of Cu NPs, respectively (Figure 1A). At the end of incubation (96 h), as much as 71.1, 48.7, and 10.7% were still motile in the control, 1 and in 5 mg Cu·L^−1^ as Cu NPs, respectively.

During the incubation of spermatozoa with CuO NPs, the earliest decrease in MOT was observed after 48 h of storage in 50 Cu mg·L^−1^ (IC_50_). After 72 h of incubation, a significant decrease in MOT was observed in 25 mg Cu·L^−1^ as CuO NPs and in 10 mg Cu·L^−1^ at 96 h. Until the end of storage motility in 1 and 5 mg Cu·L^−1^ as CuO NPs did not differ from the parameter in the control sample (Figure 1B). At 96 h of incubation, as much as 71.1, 64.1, 51.3, 38.4, 39.0, and 4.2% were still motile in the control and in 1, 5, 10, 25, and 50 mg Cu·L^−1^ as CuO NPs, respectively.

In an ionic solution of copper (CuSO_4_), a significant decrease in MOT was observed after 24 h of incubation in 25 and 50 mg Cu·L^−1^ as CuSO_4_ (IC_50_ in 50 mg Cu·L^−1^). In lower Cu concentrations, 5 and 10 mg Cu·L^−1^ as CuSO_4_, MOT decreased at 48 h of incubation. After 96 h of incubation, a significant decrease in MOT was observed at concentrations of 1 mg Cu·L^−1^ CuSO_4_ compared to the control. The cessation of movement occurred in 48, 72 and 96 h in a solution of 50, 25 and 10 mg Cu·L^−1^ as CuSO_4_, respectively (Figure 1C). At the end of incubation (96 h), as much as 71.1, 52.2, and 5.2% in the control, 1 and in 5 mg Cu·L^−1^ as CuSO_4_, respectively, were still motile (Figure 1C).

### 3.2. Curvilinear Velocity (VCL)

Curvilinear velocity (VCL) of spermatozoa stored in Cu NPs, CuO NPs and CuSO_4_ was significantly influenced only by incubation time (Table 2). Generally, a slight increase in velocity at activation in copper products was observed compared to the control, particularly up to 24 h of storage. A parallel decrease in VCL with increasing incubation time in all environments containing tested products was observed.

**Figure 2:**
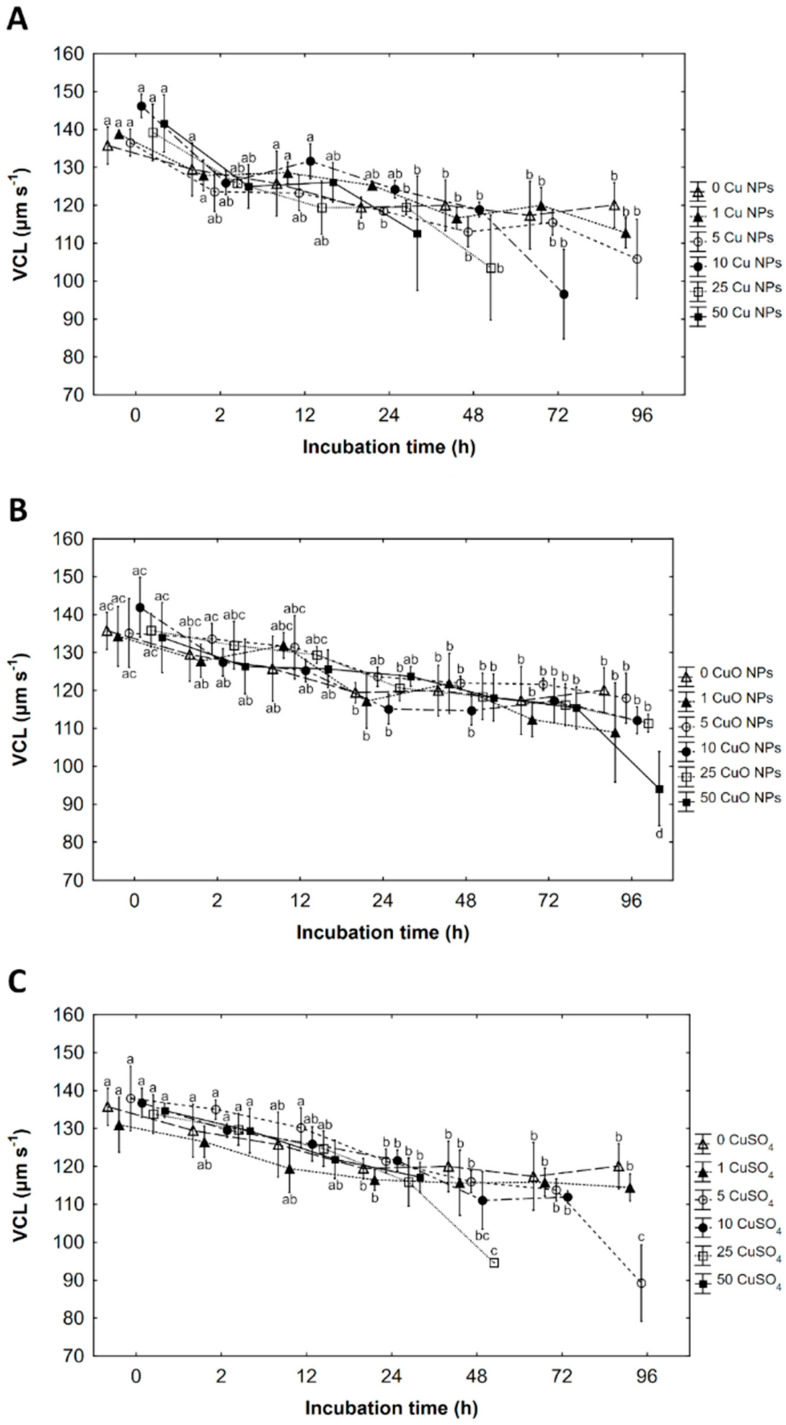
The effects on curvilinear velocity (VCL) of copper as (**A**) Cu nanoparticles (Cu NPs), (**B**) CuO nanoparticles (CuO NPs), and (**C**) ions of CuSO_4_ solution at concentration: 0, 1, 5, 10, 25 and 50 mg copper L^−1^ on rainbow trout (*Oncorhynchus mykiss* W.) spermatozoa after a defined time of incubation 0, 2, 12, 24, 72 and 96 hours (h). Sperm was mixed with incubation solution in the of ratio 1:10. Values marked with the same letter are not significantly different from one another (*p* > 0.05). Two-way or one-way ANOVAs and NIR tests were used for the post-hoc comparison. Mean value ± SEM.

At time 0, the curvilinear velocity of spermatozoa in the control was around 138 µm s^−1^ after temporary contact of spermatozoa with ASP then activated with buffered water. In solutions containing Cu NPs, nanoproduct slightly positively influenced the velocity as the parameter was slightly higher than in the control. A maximum value of 146 µm s^−1^ was achieved in 10 mg Cu·L^−1^ as Cu NPs at time 0. After 24 h of storage in most solutions velocity dropped and maintained at similar levels (115–120 µm s^−1^) until the end of storage (apart from the measurement of velocity one day before cessation of movement) (Figure 2A).

The nanoproduct containing CuO NPs slightly increased velocity at activation compared to the control up to 24 h of storage (Figure 2B). During incubation in the CuO NPs velocity decreased, particularly up to 24 h of storage, then there was little change in velocity across further storage (86–122 µm s^−1^).

In an ionic solution of CuSO_4,_ concentration of 5 mg Cu·L^−1^ at time 0 and up to 24 h of incubation, exerted a slightly positive influence on velocity compared to the control (Figure 2C). After 24 h of storage, the velocity of spermatozoa dropped to around 120 µm s^−1^, ranging between 111–121 µm s^−1^ until the end of storage (apart from the measurement of velocity one day before cessation of movement) (Figure 2C).

### 3.3. Linearity (LIN)

**Figure 3:**
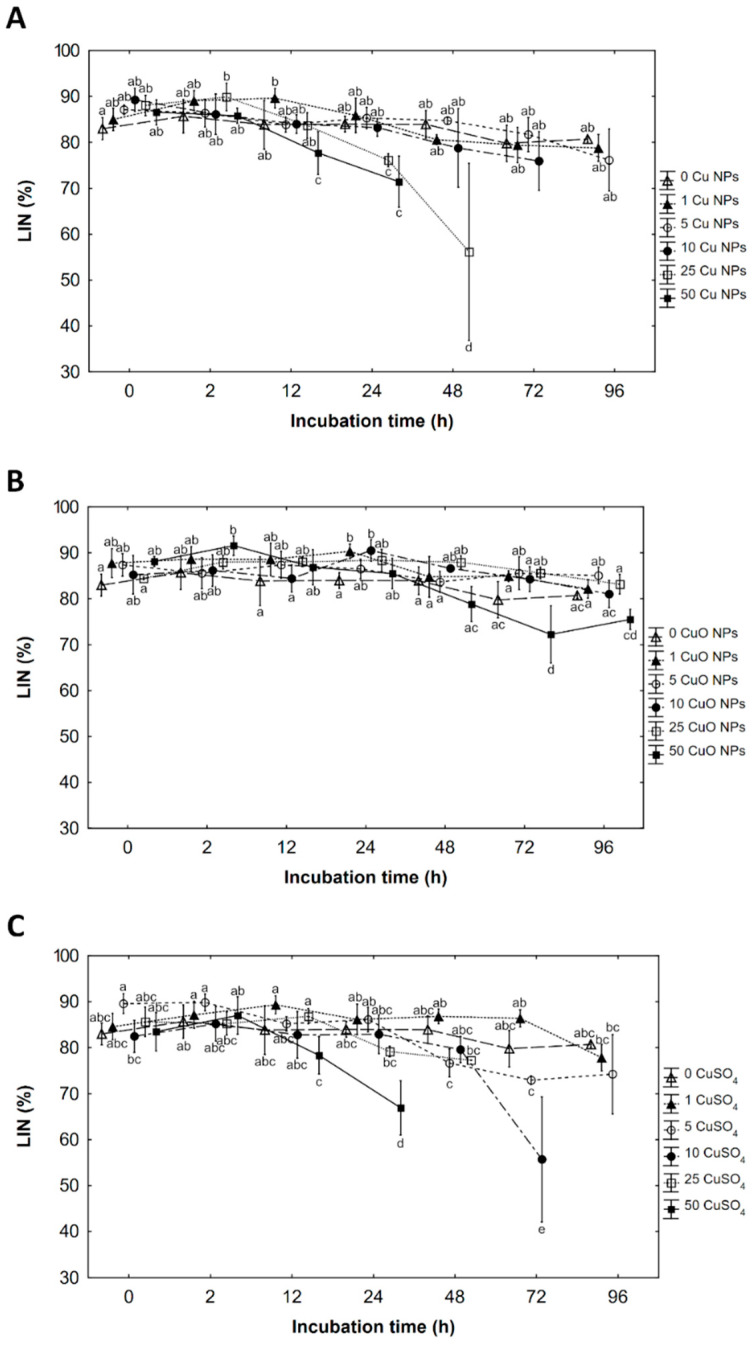
The effects on linearity (LIN) of copper as (**A**) Cu nanoparticles (Cu NPs), (**B**) CuO nanoparticles (CuO NPs), and (**C**) ions of CuSO_4_ solution at concentration: 0, 1, 5, 10, 25 and 50 mg copper L^−1^ on rainbow trout (*Oncorhynchus mykiss* W.) spermatozoa after a defined time of incubation 0, 2, 12, 24, 72 and 96 hours (h). Sperm was mixed with incubation solution in the of ratio 1:10. Values marked with the same letter are not significantly different from one another (*p* > 0.05). Two-way or one-way ANOVAs and NIR tests were used for the post-hoc comparison. Mean value ± SEM.

The interaction between the concentration of Cu NPs and incubation time was found in the linearity (LIN) (Table 2). Some concentration of Cu NPs had a slightly positive effect on LIN up to 24 h of incubation apart from the measurements before cessation of movement when a significant decrease in LIN in 25 and 50 mg Cu·L^−1^ was observed (Figure 3A).

Linearity depended on the concentration of CuO NPs and incubation time. Generally, CuO NPs have a positive or slightly positive effect on LIN ahead incubation, except for 50 mg Cu·L^−1^ as CuO NPs which causes a decrease in LIN at 72 and 96 h of incubation (Figure 3B).

A significant interaction between CuSO_4_ concentration and incubation time was found in the linearity analysis. From 0 to 24 h of incubation, concentrations of 1 and 5 mg Cu·L^−1^ as CuSO_4_ had a slightly positive effect on LIN (in the first concentration prolonged until 72 h of incubation). In some concentrations of CuSO_4_ LIN decreased a day before cessation of movement (Figure 3C).

### 3.4. Amplitude of Lateral Head Displacement (ALH)

ALH was not influenced by the concentration of Cu NPs or CuO NPs and incubation time, so the parameters fluctuated across incubation time. In CuSO_4_ solutions, ALH was only affected by the incubation time (Table 2).

### 3.5. Beat Cross Frequency (BCF)

In an ionic solution of CuSO_4_ the BCF was not influenced by the copper and incubation time. However, in NPs solutions the interaction was found between the NPs concentration and incubation time for the BCF (Table 2). The parameter increased in the highest concentration of CuO NPs up to 24 h of incubation, then decreased with increasing concentration of CuO NPs ahead storage. In Cu NPs the parameter was high in the highest concentrations of the NPs up to 2 h of incubation while after 72 h of storage a significant decrease in BCF occurred.

### 3.6. Motility Duration (MD)

Motility duration was affected by Cu NPs concentration and incubation time (Table 2). Incubation of sperm with Cu NPs slightly or significantly prolonged motility duration, particularly in time 0.

An interaction between CuO NPs concentrations and incubation time was noted on MD. The slightly positive effect of the nanoproducts on MD was noticed across the incubation time, while a significant effect was obtained at time 0.

The motility duration depended on CuSO4 concentration and incubation time, without interaction between these factors. Generally, the duration of motility was positively affected by CuSO_4_.

Some exception of the tendency was obtained in measurements at 2 and 48 h.

## 4. Discussion

Numerous studies indicated the influence of environmental contamination on fish spermatozoa mainly focused on the influence of the ionic form of heavy metals on motility and fertilisation [41,49,50,51,52,53,54,55,56,57,58,59,60,61]. So far, few studies have been conducted using nanometals. One of the earliest studies on the effects of nanometals on the motility of fish sperm was a study by Ozgur and coworkers [45,46,47,48]. The authors investigated various nanometals, concentrations, and exposure times. In Ozgur et al. [48], sperm were incubated with the oxygenated form of a nanozinc (ZnO NPs). This study concerning 4 h incubation of *Cyprinus carpio* spermatozoon in ZnO NPs (at concentrations: (0.001, 0.01, 0.1, 0.5, 1 mg·L^−1^) showed a statistically significant increase in velocity (VCL) in the lowest dose of 0.001–0.01 mg·L^−1^ ZnO NPs while it significantly decreased in concentrations higher than 0.05 ppm. The EC_50_ value for VCL was 0.56 mg·L^−1^ ZnO NPs. In our study on the rainbow trout gametes, a slightly positive effect of copper products on velocity (VCL) was observed particularly in the initial hours of incubation. During prolonged storage, the velocity in testing factors was similar to that of the control. Reduced VCL occurred in the highest concentration of copper one day before motility cessation. In our previous study on direct effect of copper nanoproducts on spermatozoa only a decrease in the VCL was noted [23]. The different result obtained in this study was due to the higher concentration of the test compounds and the different experimental protocol used. In Kowalska-Góralska et al. [23], the copper products that were diluted in activation media (buffered water) and contaminants only affected the sperm during movement. In this study the ionic form had a detrimental effect from the concentration of 8 mg Cu·L^−1^. Nanometals were less harmful than the ionic form which significantly decreased VCL from 50 mg Cu·L^−1^. In another study, Ozguretal [47] valuated the effect of different doses of Fe_3_O_4_ NPs after 24 h of incubation on the sperm kinematics of rainbow trout. Based on the results from Ozgur et al. [47], there was a statistically significant reduction in sperm velocity (VCL) after applying 50 mg·L^−1^ of Fe_3_O_4_ NPs. The same concentration of silica nanoparticles (SiO_2_ NPs) increased VCL [62]. In the Ozgur et al. [46] paper, incubation with TiO_2_ NPs for 3 h had no significant effect on VCL while decreasing VAP occurred at concentrations equal to or greater than 10 mg·L^−1^ of TiO_2_.

In a study by Ozgur et al. [46] where *O. mykiss* sperm was incubated with TiO_2_, LIN value increased up to 1 mg·L^−1^. The highest LIN occurred at 0.1 mg·L^−1^. In the Ozgur et al. study [62], incubation with SiO_2_ decreased LIN. In our study after 2–24 h of incubation, LIN values were significantly or slightly positively influenced by copper nanoparticles and low concentrations of ionic form. A reduction of LIN occurred only some days before motility cessation in a solution with the highest concentrations of copper nanoparticles.

In study by Ozgur et al. [45,46,47,48,62] the ALH was not affected by nanoproducts while the BCF increased in the highest concentrations of nanoproducts (when the short incubation time is considered). In our study, the factors tested also had no effect on ALH, while BCF increased and then decreased with increasing concentration of nanometals and extended incubation time. Generally, copper ions and nanoproducts positively influenced the time of motility duration in a specific range of concentrations similar to a previous study [23].

In the studies by Ozgur et al. [45,46,47,48,62], the percentage of motile sperm was not analysed. In the present study where rainbow trout spermatozoa were incubated with copper nanoproducts, lesser toxicity of CuO NPs than Cu NPs was seen. The effect of Cu NPs was similarly harmful to Cu ions which were more negative than CuO NPs. The reduction of MOT in the level of IC_50_ was achieved in 24, 24 and 48 h of incubation for 50 mg Cu·L^−1^ as Cu NPs, CuSO_4_ and CuO NPs, respectively. In concentrations of Cu NPs and CuSO_4_, cessation of movement occurred after 48 h of incubation, while in CuO NPs, the movement was recorded until the end of the experiment. These results confirm those obtained by Kowalska-Góralska et al. [23] that nanoproducts showed a less harmful effect on sperm motility than Cu ions but also achieved differentiated effects of nanometals. This study revealed a lower toxicity of CuO nanoparticles than that of Cu nanoparticles. Greater harmfulness of copper ions than nanoproducts was also confirmed in the studies on *Daphnia magna* in which LC_50_ for copper ions were 0.019 mg·L^−1^ [20], 0.00653 mg·L^−1^ [18], 0.045 mg·L^−1^ [17] and 0.07 mg·L^−1^ [16] when the LC_50_ for nanocopper (as CuO NPs) was 3.2 mg·L^−1^ [19], 5.9 mg·L^−1^ [26] and 6.62 mg·L^−1^ [25] after 48 h of incubation. Similar to our results CuO NPs was less harmful than Cu NPs and other metals on organisms [24,35,63,64]. However, nanometals are less harmful than ionic metals [16,17,18,19,20] while some studies have found the opposite effect [5,21,26,35,38,65].

A similar disfunction in the parameters of sperm motility was reported when the effect of heavy metal was studied as a direct effect or during incubation [49,50,51,52,53,54,55,56,57,58,59,60,61]. As well as a decrease in motility, changes in swimming velocity in activated fish sperm caused by metal contaminants are frequently noted to occur, and these variations depend on the species, the substances tested and their concentration [51,53]. Of the four species studied by Lahnsteiner et al. [53], the African catfish gametes were the most resistant to trace metal ions during direct sperm activation. Spermatozoa of chub and burbot showed medium resistance, while spermatozoa of brown trout were the most sensitive to these contaminants.

## 5. Conclusions

Nanoproducts made from different compounds of the same elements have a different effect on cells. Cu NPs were more harmful than CuO NPs. The effect of Cu NPs was similar to the ionic form of CuSO_4_. The effect of nanoproducts depends on the concentration of the substance in the environment, other features of the milieu, and the contact time with the cells. When incubated, generally copper nanoproducts and ionic form in low concentration exert a slightly positive effect on spermatozoa velocity, linearity, and motility duration, particularly up to 24 h of storage. The harmful effects of CuSO_4_, Cu NPs, and CuO NPs on the percentage of motile sperm (IC_50_) were detected after 24, 24, and 48 h, respectively, when incubated in a concentration of 50 mg Cu·L^−1^. Further studies on the effects of nanoparticles on various cells and organisms should be continued to understand the toxicity of anthropogenic products, harmful doses, and impact mechanisms.

## Figures and Tables

**Table 1:** Nanocopper toxicity to organisms shown as LC_50_—median lethal concentration values.

Organism	Nanoparticle	LC_50_	Incubation Time	Source
*Daphnia pulex*	Cu	0.51 mg·L^−1^	48 h	[22]
	CuO	0.06 mg·L^−1^	48 h	[24]
*Daphnia magna*	CuO	7.85 mg·L^−1^	24 h	[25]
	CuO	3.2 mg·L^−1^	48 h	[19]
	CuO	5.9 mg·L^−1^	48 h	[26]
	CuO	3.3 mg·L^−1^	48 h	[16]
	CuO	2.56 mg·L^−1^	24 h	[27]
	CuO	0.99 mg·L^−1^	48 h	[28]
	CuO	5.66 mg·L^−1^	48 h	[29]
	CuO	6.62 mg·L^−1^	48 h	[25]
*Thamnocephalus platyurus*	CuO	9.80 mg·L^−1^	24 h	[30]

**Table 2:** Effect of different copper products (Cu NPs, CuO NPs, CuSO_4_) and storage time on motility parameters of rainbow trout (*Oncorhynchus mykiss* W.) spermatozoa.

Dependent Variable	Concentration of Substance	*p*	Incubation Time	*p*	Interaction	*p*
Cu NPs						
MOT					F_15,75_ = 5.90	*p* < 0.001
VCL	F_5,25_ = 0.89	*p* > 0.05	F_3,15_ = 5.82	*p* < 0.01	F_15,75_ = 0.59	*p* > 0.05
LIN					F_15,75_ = 2.21	*p* < 0.05
ALH	F_5,25_ = 1.90	*p* > 0.05	F_3,15_ = 0.88	*p* > 0.05	F_15,75_ = 1.06	*p* > 0.05
BCF					F_15,75_ = 2.39	*p* < 0.01
motility duration	F_5,25_ = 5.49	*p* < 0.01	F_3,15_ = 4.00	*p* < 0.05	F_15,75_ = 1.35	*p* > 0.05
CuO NPs						
MOT					F_30,150_ = 2.27	*p* < 0.001
VCL	F_5,25_ = 1.76	*p* > 0.05	F_6,30_ = 4.04	*p* < 0.01	F_30,150_ = 0.81	*p* > 0.05
LIN	F_5,25_ = 3.64	*p* < 0.05	F_6,30_ = 2.88	*p* < 0.05	F_30,150_ = 1.39	*p* > 0.05
ALH	F_5,25_ = 1.99	*p* > 0.05	F_6,30_ = 0.39	*p* > 0.05	F_30,150_ = 1.08	*p* > 0.05
BCF					F_30,150_ = 1.58	*p* < 0.05
motility duration					F_30,150_ = 1.66	*p* < 0.05
CuSO_4_						
MOT					F_15,75_ = 5.98	*p* < 0.001
VCL	F_5,25_ = 0.81	*p* > 0.05	F_3,15_ = 5.62	*p* < 0.01	F_15,75_ = 0.14	*p* > 0.05
LIN					F_15,75_ = 1.96	*p* < 0.05
ALH	F_5,25_ = 2.58	*p* > 0.05	F_3,15_ = 6.35	*p* < 0.01	F_15,75_ = 1.44	*p* > 0.05
BCF	F_5,25_ = 1.12	*p* > 0.05	F_3,15_ = 3.22	*p* > 0.05	F_15,75_ = 1.51	*p* > 0.05
motility duration	F_5,25_ = 4.47	*p* < 0.01	F_3,15_ = 3.35	*p* < 0.05	F_15,75_ = 1.51	*p* > 0.05

Two-way repeated-measures ANOVA on the effects of copper and storage time on motility parameters of rainbow trout spermatozoa: motility percentage (MOT), curvilinear velocity (VCL), linearity (LIN), amplitude of lateral head displacement (ALH) and beat cross frequency (BCF).

## Data Availability

Not applicable.
